# Mixed infection with *Burkholderia cepacia* and *Cutibacterium acnes* following subarachnoid hemorrhage surgery: a case report

**DOI:** 10.3389/fmed.2026.1815175

**Published:** 2026-05-13

**Authors:** Lina Yao, Chenjie Zhou, Aixiang Wu, Ye Fu, Huajun Wang

**Affiliations:** 1 Department of Intensive Care Unit, The Affiliated People's Hospital of Ningbo University, Ningbo, China; 2 Department of Pharmacy, The Affiliated People's Hospital of Ningbo University, Ningbo, China

**Keywords:** antimicrobial therapy, *Burkholderia cepacia*, *Cutibacterium acnes*, mixed infection, subarachnoid hemorrhage

## Abstract

Intracranial mixed infection represents a rare and critical clinical scenario associated with high mortality and multi-organ failure, and its management remains extremely challenging. We report a 56-year-old female with chronic hepatitis B and intracranial hemorrhage, who developed a severe mixed infection with *Burkholderia cepacia* (*Burkholderia cepacia*) and *Cutibacterium acnes* (*C. acnes*). Notably, *B. cepacia* was isolated from blood and sputum specimens, whereas *C. acnes* was detected in cerebrospinal fluid. The patient experienced progressive systemic deterioration with high fever and multiple organ dysfunction. Multidisciplinary supportive care and targeted antimicrobial strategy achieved effective infection control and gradual recovery of hepatic and renal function. Nevertheless, irreversible neurological damage resulted in persistent coma, and the patient was discharged for palliative care 3 months later. This case highlights the therapeutic difficulties of severe mixed infections with intracranial involvement and provides clinical experience for the management of similar complex critical illnesses.

## 1 Introduction

*Burkholderia cepacia* (*B. cepacia*), a Gram-negative bacillus, is widely found in soil and plant root systems. Clinically, it is recognized as a rare but severely pathogenic bacterium that can cause a range of infections, including pulmonary infections, bloodstream infections, and urinary tract infections (1, 2). *B. cepacia* intracranial infection is one of the complications seen in severely ill patients who often have underlying diseases or immunosuppression. Due to the diverse causes of infection and antibiotic resistance, treatment poses significant challenges, and therapeutic strategies are highly complex and varied (3). Compared to other *Burkholderia* species, *B. cepacia* intracranial infection has a higher mortality rate. *Cutibacterium acnes* (*C. acnes*) is a component of the normal human microbiota, and only a very small proportion of cases give rise to acute infection (4, 5). Multiple studies have demonstrated that this bacterium is a causative agent of various implant-associated infections, including those related to breast implants, neurosurgical shunts, and spinal surgical procedures (5). In recent years, the incidence of postoperative neurosurgical infections secondary to *C. acnes* has increased, with such infections presenting with non-specific clinical manifestations (6).

This study reports a case of a patient with subarachnoid hemorrhage (SAH) who was in a deep coma and had no spontaneous breathing. After the implantation of an Ommaya reservoir, the patient’s condition rapidly worsened and developed into multiple organ dysfunction syndrome (MODS). The patient was later diagnosed with a mixed intracranial infection caused by *B. cepacia* and *C. acnes*. With timely plasma exchange, continuous renal replacement therapy (CRRT) for organ support, and targeted anti-infective treatment, the patient’s condition improved significantly, and the patient was eventually discharged in a stable clinical state.

## 2 Case presentation

**Figure 1:**
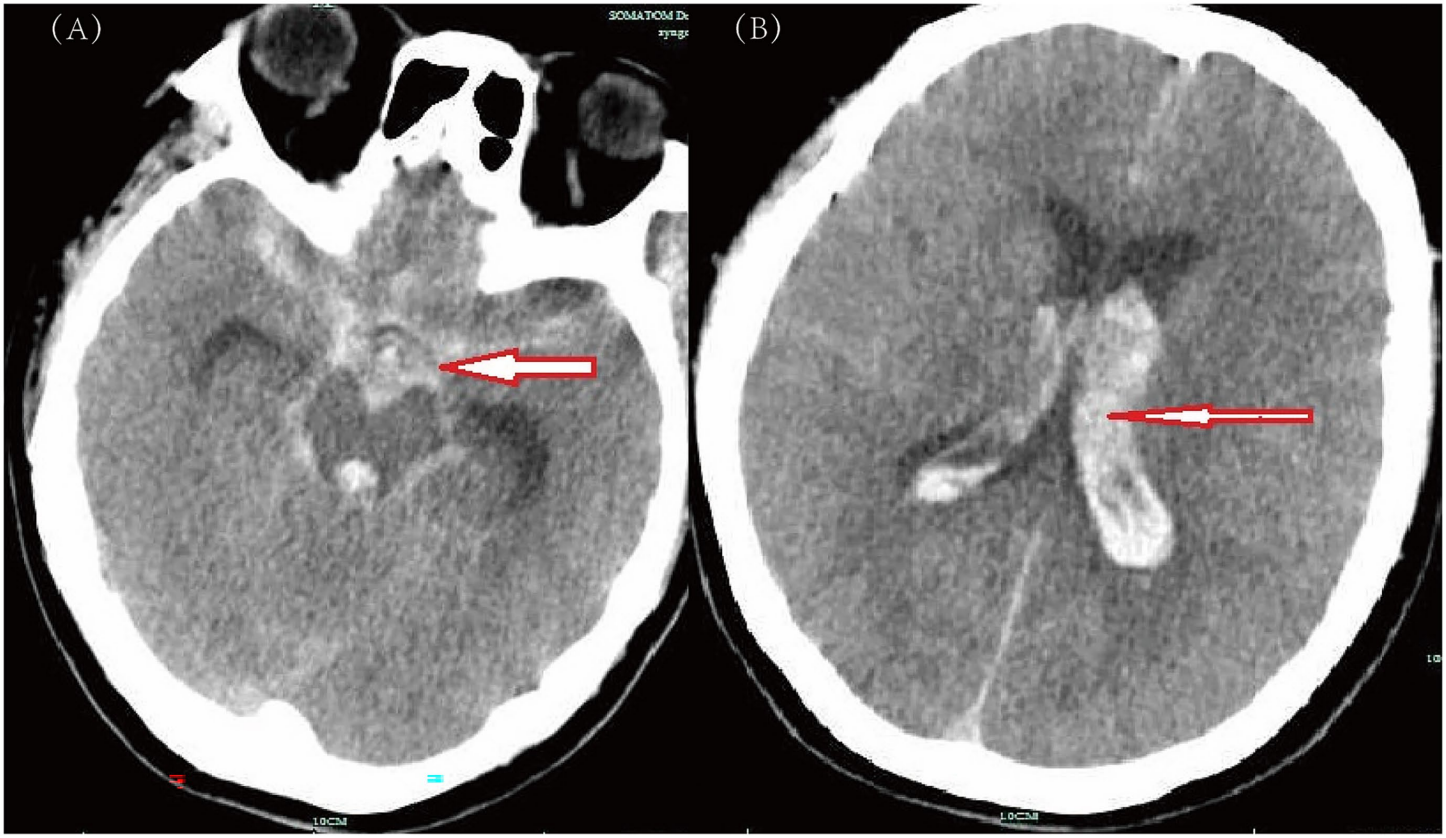
Computed tomography (CT) scans of the brain obtained on admission. **(A)** Axial view showing effacement of cerebral sulci and gyri, with hyperdense areas in the basal cisterns (red arrow) consistent with subarachnoid hemorrhage. **(B)** Axial view at a higher level demonstrating intraventricular extension of hemorrhage (red arrow) and compression of the bilateral lateral ventricles, indicating severe subarachnoid hemorrhage complicated by intraventricular hemorrhage and mass effect.

**Figure 2:**
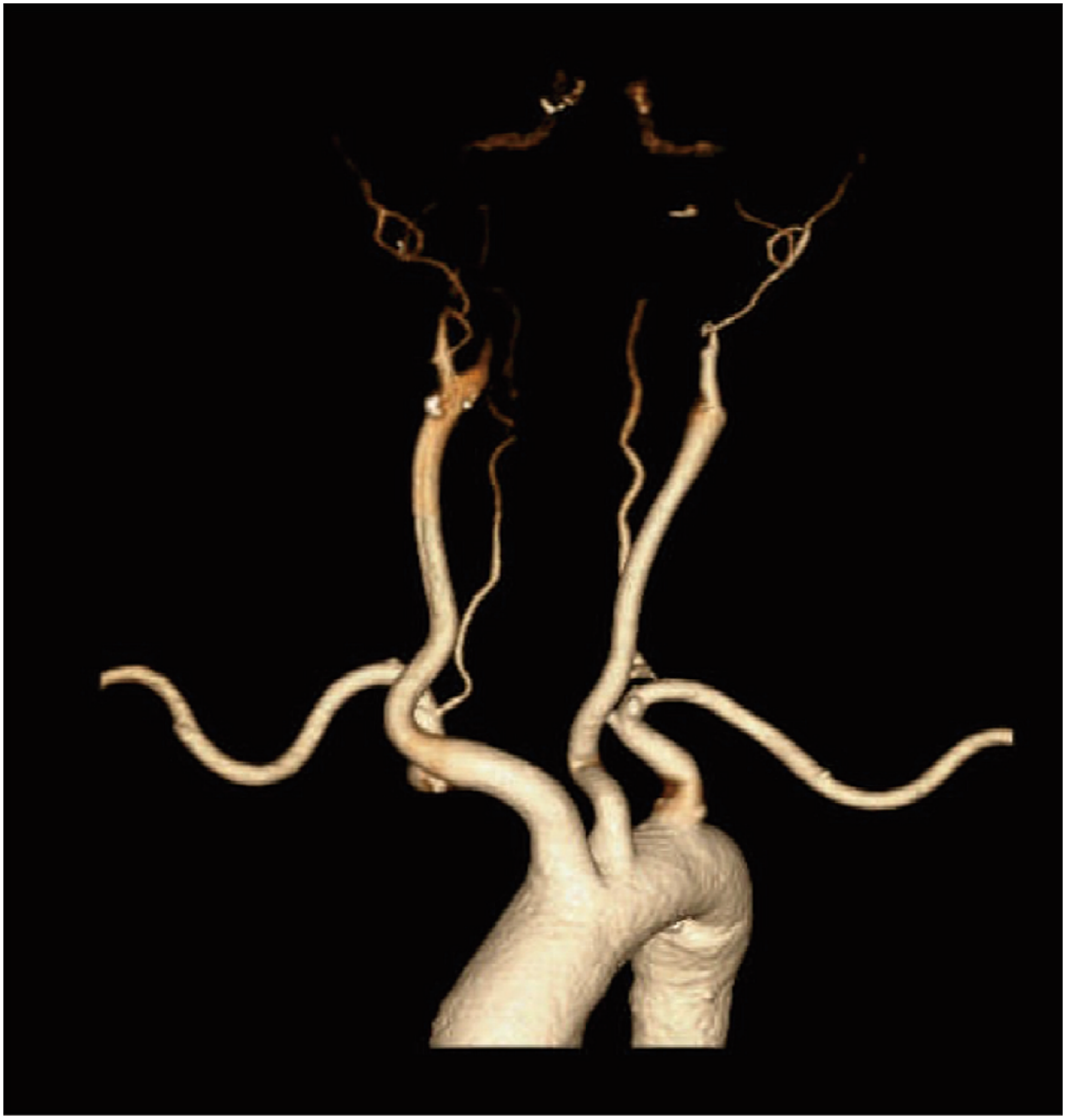
The head CT angiography (CTA) examination reveals non-opacification of bilateral distal carotid arteries, indicating intracranial hypertension.

A 56-year-old female patient with a 30-year history of chronic hepatitis B (HBsAg, HBeAb, and HBcAb positive) presented to the emergency department of our hospital, after suddenly losing consciousness at home on November 15, 2023. Her family members immediately performed chest compressions for over 10 min until the emergency ambulance arrived. Upon admission, the patient presented with critical symptoms such as deep coma, absence of spontaneous respiration, dilated pupils on both sides, and disappearance of large artery pulsations. The emergency department promptly performed 6 minutes of chest compressions and endotracheal intubation as rescue measures. Thirty minutes after cardiopulmonary resuscitation (CPR), the patient’s vital signs were relatively stable. A head CT scan was then performed with ventilator support and administration of high-dose vasoactive drugs, which revealed SAH (Figure 1). One hour later, during transport to the ICU, a Computed tomography angiography (CTA) was performed, which revealed non-visualization of the cerebral vasculature (Figure 2). However, the patient suffered a recurrent cardiac arrest, received 3 minutes of chest compressions, and subsequently regained spontaneous circulation. The family was informed of the grave prognosis and the high risk associated with surgical intervention. Nonetheless, the Ommaya reservoir implantation was performed on the day of admission at the family’s insistent request.

**Figure 3:**
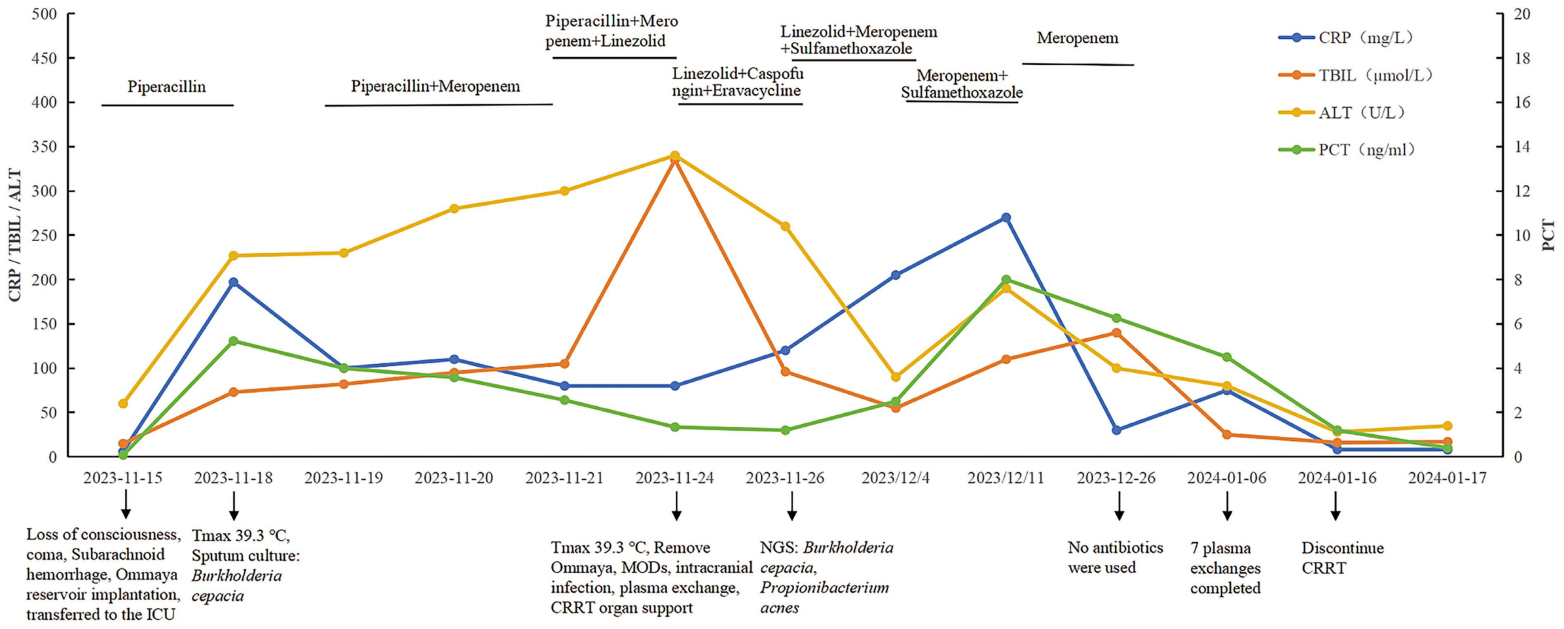
Clinical course of the patient.

**Figure 4:**
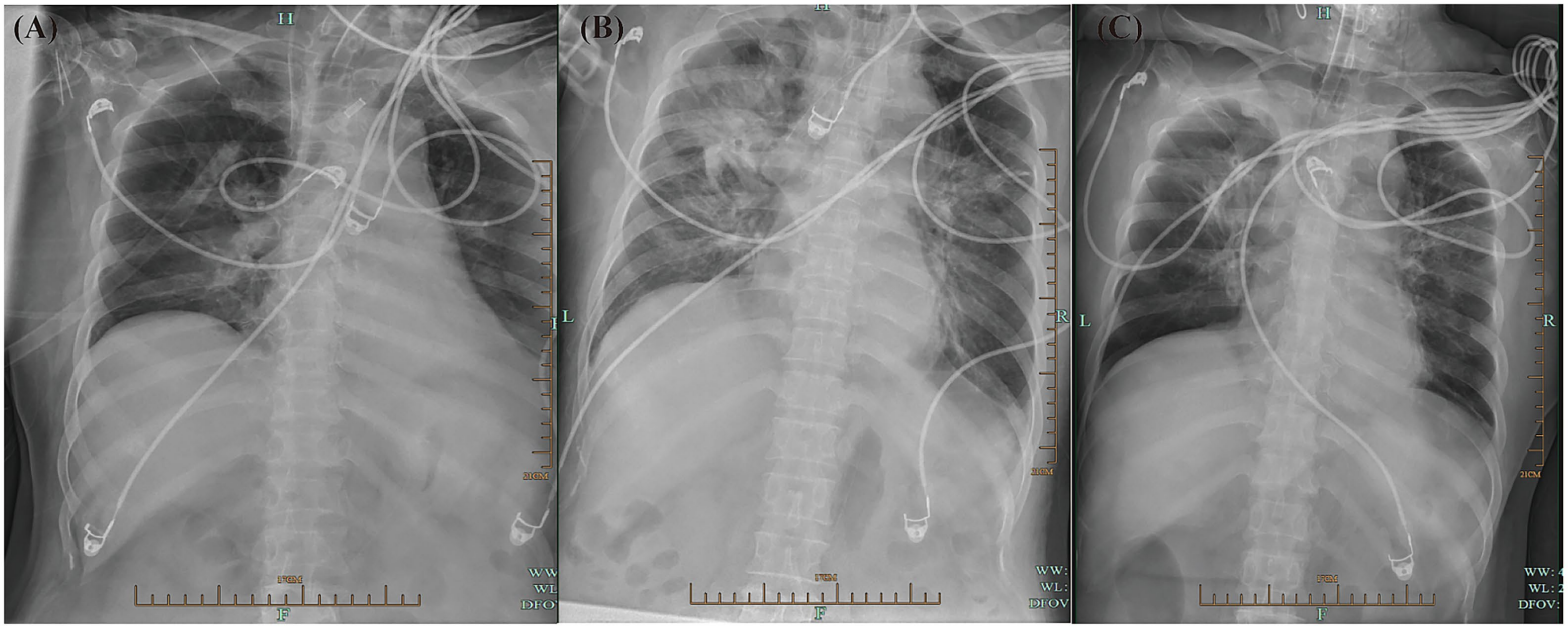
Serial bedside chest radiographs illustrating dynamic pulmonary changes during hospitalization. **(A)** Day 2: Mild bilateral peribronchial and interstitial infiltrates. **(B)** Day 5: Marked progression to extensive bilateral pneumonia, with diffuse alveolar and interstitial opacities and prominent right lung consolidation. **(C)** Day 40: Significant resolution of pulmonary infiltrates and improved bilateral lung aeration.

On the day of ICU admission, the patient’s white blood cell count (WBC) was 15.2 × 10^9^/L, with a neutrophil percentage of 33.4%. Her C-reactive protein (CRP) level was 6 mg/L, and her procalcitonin (PCT) level was 0.08 ng/mL (Figure 3). She received empirical anti-infective treatment with piperacillin-tazobactam (4.5 g, IV infusion q8h). Four days after admission (November 19), the patient’s symptoms had not improved. Her temperature was 39.3 °C, WBC was 16.9 × 10^9^/L, and the neutrophil percentage was elevated at 92.3%. At the same time, her liver function deteriorated, with aspartate aminotransferase (AST) significantly increasing to 243 U/L, alanine aminotransferase (ALT) to 227 U/L, and total bilirubin (TBIL) to 73 μmol/L. Sputum culture showed *B. cepacia* positivity. Antimicrobial susceptibility testing was performed according to the CLSI 2023 criteria, and the results showed that the cultured isolates of *B. cepacia* were susceptible to ceftazidime, meropenem, and Trimethoprim/Sulfamethoxazole (Table 1). Chest radiography revealed bilateral pulmonary infiltrates suggestive of pneumonia, and a diagnosis of bilateral pneumonia was established (Figure 4). On the fifth day of admission (November 20), the patient’s anti-infection regimen was adjusted to meropenem (1.0 g, IV infusion q8h), and piperacillin-tazobactam was discontinued, but the symptoms still did not improve. Subsequently, on the seventh day of admission (November 22), linezolid (0.6 g, IV infusion q12h) was added, yet the infection remained uncontrolled. On the tenth day after admission, the patient developed MODS involving the liver and kidneys, with a significant increase in the need for vasopressors. Therefore, on the basis of the existing anti-infective therapy, caspofungin (50 mg, IV infusion qd) and eravacycline (0.1 g, IV infusion q12h) were empirically added for broad-spectrum coverage. When subcutaneous abscess and erythema were discovered, the patient had the Ommaya reservoir removed, and vacuum-assisted closure surgery debridement and drainage (VSD) were initiated. The patient continued to have persistent high fever (38.8–39.3 °C) and showed a progressive increase in serum creatinine (peak value 252 μmol/L) and TBIL (peak value 335 μmol/L) levels, leading to the development of MODS requiring organ support therapy such as plasma exchange and CRRT. On day 12, Targeted next-generation sequencing (tNGS; Hangzhou Fanglue Biotechnology Co., Ltd.) identified *B. cepacia* in blood and sputum samples, as well as *C. acnes* in cerebrospinal fluid (CSF) (Table 2). CSF routine and biochemical profiles were consistent with intracranial infection, although CSF culture was negative (Table 3). Subsequently, Eravacycline and caspofungin were discontinued, and the patient’s anti-infection regimen was adjusted to a combination therapy of meropenem (1.0 g, IV infusion q8h), linezolid (0.6 g, IV infusion q12h), and sulfamethoxazole (0.96 g, IV infusion q12h).

**Table 1:** Antimicrobial susceptibility of *Burkholderia cepacia.*

Antibiotic	Sensitivity	Disk diffusion diameter (mm)
Amikacin	Resistant	≥64
Aztreonam	Resistant	16
Ceftazidime	Susceptible	2
Cefoperazone/Sulbactam	Resistant	16
Ciprofloxacin	Resistant	≥4
Doxycycline	Intermediate	8
Cefepime	Resistant	≥32
Imipenem	Resistant	≥16
Levofloxacin	Resistant	≥8
Meropenem	Susceptible	4
Minocycline	Intermediate	8
Ticarcillin/Clavulanic Acid	Resistant	≥128
Tobramycin	Resistant	≥16
Trimethoprim/Sulfamethoxazole	Susceptible	≤20
Colistin	Resistant	≥16
Tigecycline	Intermediate	4

**Table 2:** Test results of tNGS.

Species name	Cerebrospinal fluid sequence number (RPM)	Blood sequence number (RPM)	Sputum sequence number (RPM)
*Burkholderia cepacia* complex	—	112	4,641
*Cutibacterium acnes*	52,783	—	—

RPM, reads per million mapped reads.

**Table 3:** Cerebrospinal fluid (CSF) analysis on day 10 of admission.

Parameter	Result	Reference range	Unit
Appearance			
Color	Yellow	Colorless	-
Clarity	Clear	Clear	-
Pandy’s Test	(++) Positive	Negative	-
Red Blood Cell Count	800	0	/μL
White Blood Cell Count	163	0–5	/μL
Adenosine Deaminase	5	–	U/L
Glucose	1.77	2.22–4.50	mmol/L
Chloride	112	120–132	mmol/L
Total Protein	1304.1	150–450	mg/L
Lactate	6.8	0.5–2.2	mmol/L
Procalcitonin (PCT)	0.46	–	ng/mL
Culture	—	Negative	-

Linezolid was discontinued on day 19 (December 4) according to the etiological results, and sulfamethoxazole was withdrawn after 2 weeks (December 11). Meropenem was maintained for one month before the complete cessation of all antimicrobial agents. After antibiotic discontinuation, the patient’s body temperature and inflammatory markers returned to normal. Hepatic function gradually improved following seven sessions of plasma exchange and became nearly normal by January 6; CRRT was discontinued 2 months later on January 15. Nevertheless, given the persistent deep coma (GCS score 3 T–4 T), the patient’s family finally decided to withdraw further treatment after 3 months, with palliative care provided thereafter.

## 3 Discussion and conclusions

*Burkholderia cepacia* is a Gram-negative opportunistic pathogen that often causes widespread infections in immunocompromised individuals. *B. cepacia* can survive and replicate in invasive devices, making it an important nosocomial pathogen (2, 7). In addition, *B. cepacia* has unique antimicrobial characteristics, especially resistance to polymyxins and aminoglycoside antibiotics, which also poses significant challenges to treatment (8). Previous studies have also found that co-infection with multi-drug resistant *B. cepacia* and SARS-CoV-2 may exacerbate inflammation and cause severe lung damage (9). Our study confirmed similar results, with multi-drug resistant *B. cepacia* causing bilateral pneumonia, and the patients rapidly progressing to MODS within a few days. *B. cepacia* may invade the bloodstream and CSF via respiratory colonization or surgical site contamination, whereas *C. acnes*, a skin commensal organism, is consistent with the typical characteristics of neurosurgical device-related infection (9–11). The growth of *C. acnes* is characteristically slow, and conventional cultures frequently produce false-negative results (10). Therefore, NGS can provide etiological evidence of infection in a timelier manner (10). Several case series have demonstrated that *C. acnes* ranks as the second most common pathogen in postoperative intracranial infections (11). As a constituent of normal human skin flora, this organism can directly invade intracranial tissues during neurosurgical procedures. In the present case, the patient received urgent Ommaya reservoir implantation on the day of admission and subsequently developed severe intracranial infection (10). The surgical placement of the Ommaya reservoir was the most likely route for secondary *C. acnes* intracranial infection. The synergistic pathogenicity of these two pathogens exacerbates the severity and persistence of infections, particularly as *B. cepacia* in onions possesses intrinsic multidrug resistance, often leading to failure of empirical antimicrobial therapy (12–14). Meanwhile, *C. acnes* can form biofilms on the surface of the Ommaya reservoir, preventing antibiotics from reaching effective bactericidal concentrations and resulting in persistent infection. Previous studies have shown that *C. acnes* accounts for 9 to 25% of neurosurgical postoperative infections, and approximately 41% of these cases present as polymicrobial mixed infections (12). Furthermore, the severe systemic inflammatory response induced by mixed infection further caused hepatic and renal dysfunction and multiple organ dysfunction, forming a vicious cycle of infection–organ dysfunction–worsening infection.

Severe intracranial hypertension also reduced the central nervous system penetration of antimicrobial agents, further rendering the infection refractory to treatment. The pharmacokinetic /pharmacodynamic properties of antimicrobial agents and their central nervous system penetration are critical in the treatment of intracranial infections, and are particularly important in patients receiving continuous renal replacement therapy. However, its resistance rates to meropenem, sulfamethoxazole/trimethoprim, and semi-synthetic penicillins are currently low. In this case, the strain is susceptible to meropenem. A dosage of 1.0 g intravenously every 8 h meets the pharmacokinetic/pharmacodynamic (PK/PD) targets for time-dependent bactericidal activity against Gram-negative bacilli. No additional dose adjustment is required under conventional CRRT modalities, and effective CSF concentrations can be ensured. However, in recent years, studies have shown an increasing concern regarding resistance rates. For instance, one study found that the susceptibility rate of *B. cepacia* to meropenem was only 27% (13). Hence, when the effect of monotherapy is ineffective or in cases of severe infections, combination therapy with different drugs should be considered. However, currently there are no clear guidelines recommending specific antimicrobial combinations for the treatment of intracranial infections caused by *B. cepacia* and *C. acnes*, and relevant case reports remain relatively scarce (14). Due to the presence of the blood–brain barrier, some drugs have lower concentrations in the central nervous system, such as tobramycin, or may exhibit central side effects, such as quinolones like levofloxacin. Therefore, drug selection is relatively limited when treating complex intracranial infections. Although sulfamethoxazole/trimethoprim is less commonly used in mainland China, it maintains high sensitivity against some pathogens, with studies showing a resistance rate of only 5.1% against *B. cepacia* (15). For some non-intracranial infections caused by *B. cepacia*, studies have shown that the combination of meropenem and sulfamethoxazole/trimethoprim can achieve certain therapeutic effects (16, 17). Considering their high brain tissue permeability and low resistance rates, the combination therapy is considered feasible in this clinical setting (18). Overcoming monotherapy resistance limitations. Although *B. cepacia* is intrinsically resistant to linezolid, linezolid is effective against Gram-positive bacteria such as *C. acnes* and anaerobes. Therefore, empirical use of linezolid in the early stage of infection when the pathogen is unidentified can cover *C. acnes* and other potential mixed Gram-positive pathogens, which aligns with the principle of broad-spectrum coverage for severe healthcare-associated infections. Once the pathogen is identified by tNGS caspofungin should be promptly discontinued to avoid unnecessary drug exposure and hepatorenal toxicity. The sequential adjustment of antibiotics, guided by pathogen identification, clinical efficacy, and inflammatory markers, conforms to the strategy of precision therapy for severe infections.

In addition to targeted antimicrobial therapy, multidisciplinary comprehensive supportive treatment and infection control measures are key to successful treatment. Timely removal of the Ommaya reservoir, along with VSD for debridement and drainage to eliminate the infectious focus and biofilm carrier, is a prerequisite for controlling implant-associated infections. Plasma exchange can clear inflammatory mediators, while CRRT maintains fluid, electrolyte, and acid–base balance, as well as provides renal support, thereby creating favorable conditions for antibiotics to exert their therapeutic effects. tNGS of blood, sputum, and CSF successfully detected *B. cepacia* and *C. acnes*, overcoming the limitations of traditional culture methods, which have low positivity rates and long turnaround times, and providing critical evidence for targeted anti-infective therapy. tNGS detected *B. cepacia* in sputum and blood, and combined with the clinical manifestations of systemic infection, it confirms that it is a true pathogen rather than simple colonization or contamination. The detection of *C. acnes* in cerebrospinal fluid is highly consistent with device-related central nervous system infection, supporting the diagnosis of mixed infection.

This study has several limitations. First, because *C. acnes* was detected by tNGS but not by culture, no *in vitro* antimicrobial susceptibility testing was performed for this organism, and the selection of anti-*C. acnes* agents relied primarily on clinical experience and published literature. Second, multiple interventions—including antibiotic therapy, device removal, and organ support—were implemented concurrently, making it impossible to fully differentiate the contribution of each individual measure to clinical improvement.

This report describes a rare case of mixed infection with multidrug-resistant *B. cepacia* and *C. acnes*, which ultimately progressed to MODS. Following targeted anti-infective therapy, plasma exchange, and organ support with CRRT, the patient’s clinical condition improved. This case highlights the importance of accurate microbiological identification, individualized combination antimicrobial therapy, and multidisciplinary collaboration in the prompt diagnosis and management of complex intracranial infections. The clinical improvement may be associated with the combination regimen including meropenem, linezolid, and sulfamethoxazole, as well as timely infection source control and organ support. Clinicians should maintain a high index of suspicion for mixed infections involving opportunistic pathogens such as *B. cepacia* and *C. acnes* in neurosurgical patients.

## Data Availability

The original contributions presented in the study are included in the article/supplementary material, further inquiries can be directed to the corresponding author.
